# Intravitreal and Subtenon Depot Triamcinolone as Treatment of Retinitis Pigmentosa Associated Cystoid Macular Edema

**DOI:** 10.1155/2013/591681

**Published:** 2013-12-23

**Authors:** Sidnei Barge, Renata Rothwell, Paula Sepúlveda, Luís Agrelos

**Affiliations:** Department of Ophthalmology, Vila Nova Gaia/Espinho Hospital, Rua Conceição Fernandes, 4434-502 Vila Nova Gaia, Portugal

## Abstract

We present a case of retinitis pigmentosa (RP) related cystoid macular edema (CME) refractory to oral acetazolamide and topical ketorolac that was treated with intravitreal and subtenon depot triamcinolone. A 32-year-old male with RP presented with complaints of bilateral decrease in visual acuity. His best-corrected visual acuity (BCVA) was 20/50 in the right eye and 20/100 in the left eye. After being informed of the available treatment options, the patient received bilateral intravitreal injection triamcinolone. The patient's BCVA improved to 20/40 in the right eye and 20/50 in the left eye and the CME was resorbed. However, 5 months after the injection in the left eye and two months in the right eye, visual acuity decreased due to recurrence of CME. We performed a second intravitreal injection in the left eye with improvement of visual and anatomic results, but we observed a recurrence of CME. Afterwards, we treated the patient with subtenon depot triamcinolone in both eyes, with the result that there was no recurrence after 4 months in OD or after 3 months in OS. We conclude that intravitreal and subtenon depot triamcinolone appear to provide at least temporary benefit in refractory CME as regards the improvement of visual acuity.

## 1. Background

Retinitis pigmentosa (RP) is a genetically heterogeneous group of inherited retinal dystrophies caused by progressive loss of the rod and cone photoreceptors and characterised by night blindness, peripheral visual field loss, and retinal pigment deposits visible on fundus examination [1].

Cystoid macular edema (CME) is a relatively uncommon condition in RP, with a prevalence of 10–20%. CME may markedly reduce central vision and lead to severe visual handicap in eyes already visually impaired with RP [2–4].

The treatment options for CME include laser photocoagulation, vitreoretinal surgery, topical and systemic carbonic anhydrase inhibitors, systemic corticosteroids, and intravitreal triamcinolone acetonide (IVTA). According to previous studies, the most effective therapies seem to be acetazolamide and corticosteroids [5–9].

We describe a case of CME related RP treated with intravitreal and subtenon depot injection of triamcinolone acetonide and present a review of the literature.

## 2. Case Report

**Figure 1:**
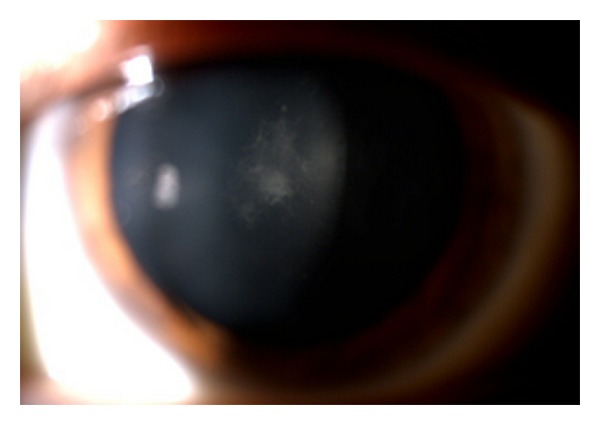
Mild subcapsular cataract in the left eye.

A 32-year-old white male was referred to our department with a diagnosis of RP and bilateral decrease of visual acuity over several years. In the previous year he had been treated for CME with oral acetazolamide (250 mg bid) and topical ketorolac (bid) without success. There was no history of systemic complications. He had a family history of RP in a paternal cousin. His BCVA was 20/50 in the right eye and 20/100 in the left eye. Biomicroscopy of the anterior segment revealed mild subcapsular cataract in the left eye (Figure 1) and was unremarkable in the right eye.

**Figure 2:**
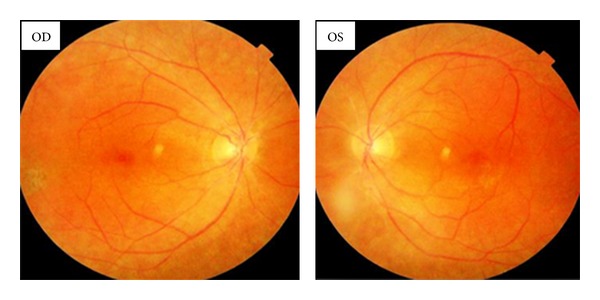
Peripheral and equatorial hyperpigmentation, arteriolar attenuation, pallor of the optic nerve, and CME.

Fundus examination revealed bilateral optic disc pallor, arteriolar attenuation, equatorial and peripheral hypopigmentation, and CME (Figure 2).

**Figure 3:**
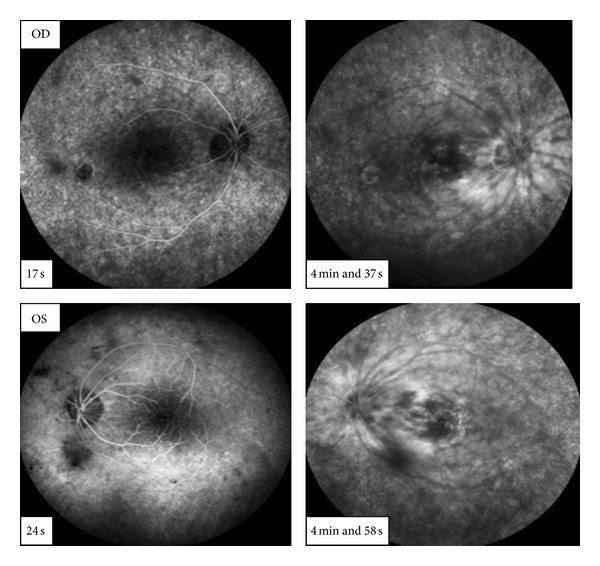
Fluorescein angiography—little accumulation of the dye in the late phases.

Fluorescein angiography (FA) showed multiple small focal fluorescein leaks and late pooling of the dye in extracellular cystoid spaces (Figure 3).

**Figure 4:**
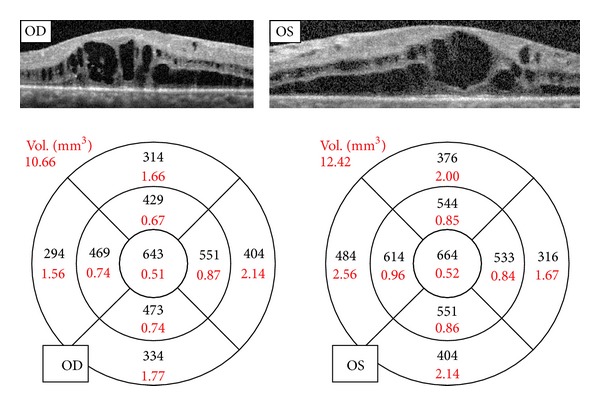
OCT spectral domain: diffuse retinal thickening with cystic areas of low reflectivity.

The optical coherence tomography (OCT) spectral domain revealed diffuse retinal thickening with cystic areas of low reflectivity (Figure 4).

**Figure 5:**
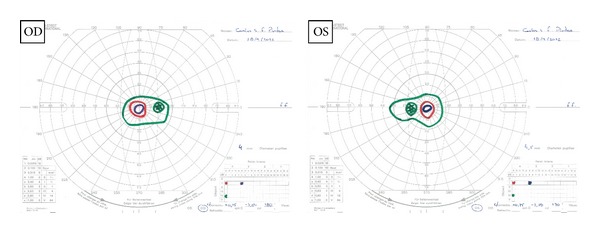
Goldmann kinetic perimetry: tubular visual field.

The Goldmann kinetic perimetry showed tubular visual field (Figure 5). Intravitreal injection of 0.1 mL of triamcinolone acetonide (40 mg/mL solution) was performed bilaterally. The left eye was treated 4 months prior to the right eye. The second intravitreal injection in the left eye was performed 9 months after the first treatment.

Subsequently, the patient was submitted to subtenon depot triamcinolone acetonide (40 mg) at 11-month (OS) and at 5-month (OD) follow-up.

**Figure 6:**
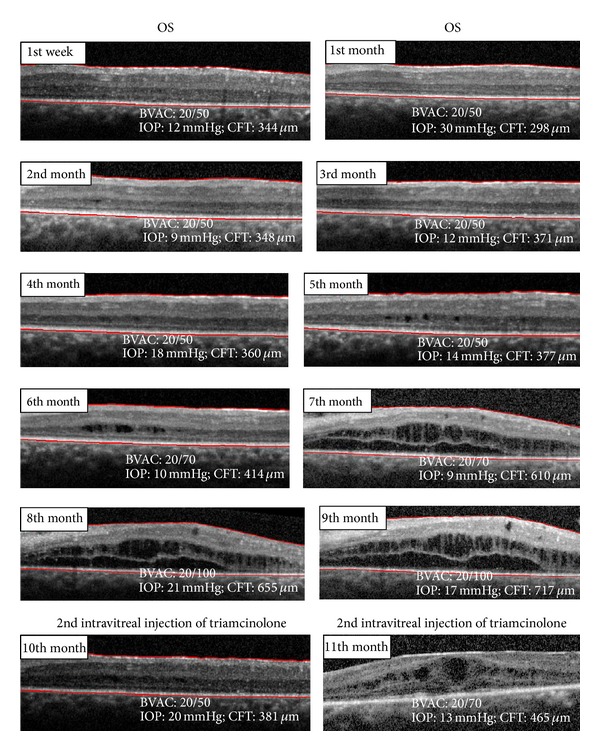
OCT spectral domain OS after 2nd intravitreal injection of triamcinolone.

One week after the first intravitreal injection, BCVA in the left eye improved to 20/50 and the OCT showed reestablishment of the foveal depression and no macular edema. After one month the patient had an increase of intraocular pressure (IOP = 30 mmHg) that was controlled with topical timolol and brimonidine. In the following 9 months there was a decrease in visual acuity and a recurrence of the CME, mainly after the 6th month. After the second intravitreal injection in the left eye, the CME and visual acuity improved but we observed a further recurrence of CME on the 11th month of follow-up (Figure 6).

**Figure 7:**
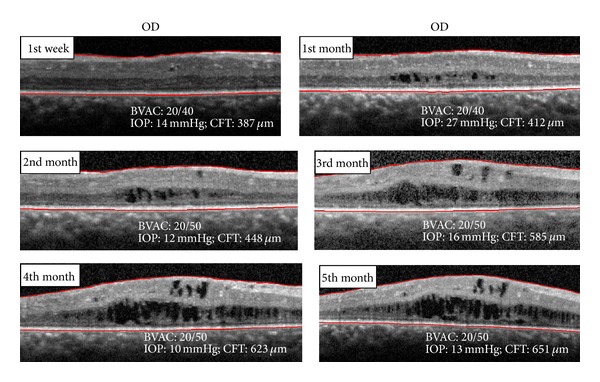
OCT spectral domain OD after intravitreal injection of triamcinolone.

In the right eye, the BCVA improved to 20/40 and the OCT revealed a decrease of the CME one week after the injection in the right eye. As in the left eye, the intraocular pressure increased (IOP = 27 mmHg) with topical timolol and brimonidine being prescribed at the first follow-up visit. The BVCA and CME worsened during the 5 months after the injection (Figure 7).

**Figure 8:**
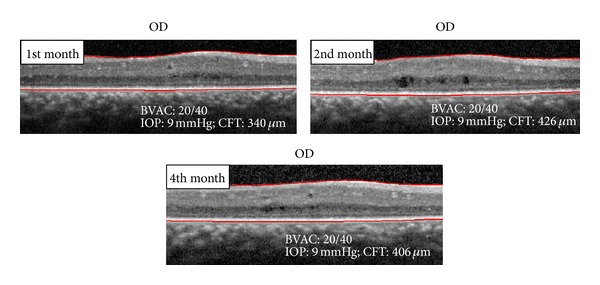
OCT spectral domain OD after subtenon depot triamcinolone.

**Figure 9:**
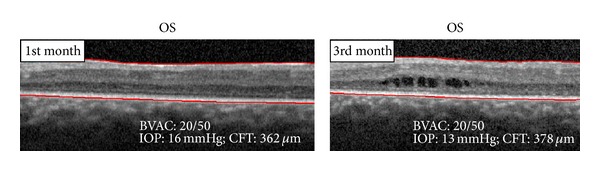
OCT spectral domain OS after subtenon depot triamcinolone.

**Figure 10:**
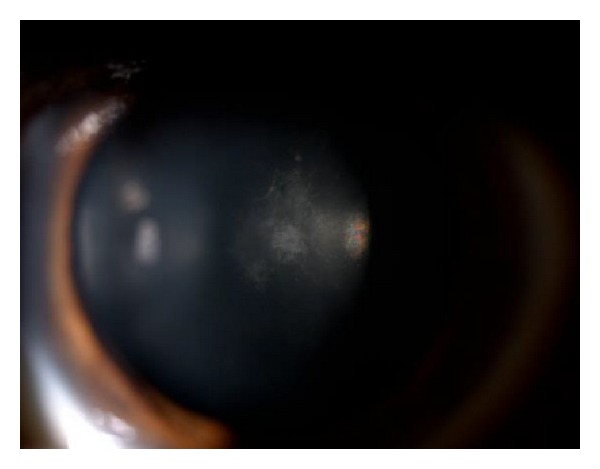
Biomicroscopy of left eye showed aggravation of subcapsular cataract.

After bilateral subtenon injection of depot triamcinolone, the CME decreased and BCVA improved (Figures 8 and 9). After three months, the patient had a further recurrence of CME and mild aggravation of the subcapsular cataract in the left eye (Figure 10). No recurrence of the CME occurred during the 4 months following the subtenon triamcinolone in the right eye.

Currently, the patient is being treated with topical timolol and brimonidine to control the intraocular pressure.

## 3. Discussion

RP is a degenerative disease characterised by pigment deposits predominantly in the peripheral retina with the central retina being relatively unaffected [10].

Typical RP is also a rod-cone dystrophy, owing to a primary progressive degeneration of the photoreceptor rods, with secondary degeneration of cones. Several signs such as RPE attenuation and bone spicule pigmentation, foveomacular atrophy, waxy pallor of the optic disk, and retinal arteriolar narrowing can occur [11].

FA has traditionally been used for the diagnosis of CME and monitoring patients. With the development of noninvasive OCT, the monitoring of patients with RP and CME can be carried out in a more delicate and noninvasive fashion [12, 13].

Hirakawa and colleagues reported the prevalence of CME in RP patients using OCT to be 13%. They also observed that OCT imaging could detect CME lesions in RP patients even in eyes with either little or no dye accumulation on FA or cystic macular lesions visible by ophthalmoscopy [12]. Stanga et al. presented preliminary findings showing that OCT imaging is at least as sensitive as FA for identifying CME and is a useful procedure for evaluating a response to therapy [13]. It is likely that FA captures current leakage activity, whereas OCT imaging reflects the accumulative effect of leakage [14].

Currently, there is no therapy that stops the evolution of RP or restores vision. The therapeutic approach is restricted to treating complications such as cataract and macular edema.

CME causes symptoms such as blurred and reduced visual acuity and subsequent atrophic changes in the fovea.

The pathogenesis of CME in RP is poorly understood and several mechanisms have been proposed to explain how CME develops.

In RP genetic defects lead to apoptosis. The resultant accumulation of metabolic by-products secondary to apoptosis disrupts retinal function and manifests as lipofuscin deposition, retinal gliosis, photoreceptor loss, choriocapillaris occlusion, and RPE hyperplasia. These RPE changes compromise the blood-retinal barrier, resulting in subretinal leakage and macular edema [15–17]. Studies have found an increased permeability of the RPE and perifoveal capillaries to fluorescein in eyes with RP [15, 17–19]. A breakdown in the blood-retinal barrier allows fluid to accumulate in cystoid spaces within the retina [20–22].

Furthermore, it has been postulated that the mechanism of CME is due to the failure of the pumping activity of the RPE, which occurs in cases characterised by later spreading of the FA staining at the level of the RPE in the late transit phases of FA [2, 23, 24]. RPE may lose polarised apical distribution in the presence of macular edema. In this condition, the RPE would be unable to effectively pump out ions and fluid from the outer retina [25].

It is postulated that carbonic anhydrase inhibitors may be exerting their therapeutic effect by restoring the polarity and hence the function of the RPE cells [25]. Studies by Fishman and colleagues and Cox and colleagues have demonstrated improvement in BCVA with oral acetazolamide sodium at a daily dose of 500 mg for patients who have RP with CME [8, 25]. However, the CME in RP patients is most often chronic and does not improve with this treatment. The adverse effects including fatigue, renal stones, loss of appetite, hand tingling, and anaemia may limit its clinical use. Topical administration of dorzolamide is ineffective [26]. Our patient received oral acetazolamide during one year without improvement of the CME, but there were no adverse effects.

Moreover, the dysfunction of anticarbonic anhydrase and enolase activity by autoantibodies in the RPE may lie at the root of edema formation. CME in RP is a negative prognostic factor and is associated with an increase of circulating antiretinal antibodies and with anatomical features that could aggravate visual recovery [27–29]. Heckenlively et al. think that a breakdown of the blood-retinal barrier during the retinal degenerative process could release possibly antigenic retinal proteins into the circulation. This could explain how retinal antigens sensitize the immune system and how antiretinal antibodies can reach the retina when normally the blood-retinal barrier would prevent this happening. However, it is not known whether antiretinal antibodies in general or only specific ones are harmful and if there are cofactors that contribute to pathogenicity [28].

The finding of this immunopathogenesis in RP has potential implications for treatment.

Steroids can produce their effect through several mechanisms, including decrease in synthesis and release levels of proinflammatory cytokines (prostaglandins and leukotrienes, vascular endothelial growth factor, and intercellular adhesion molecule 1 [30–33]), reduction in levels of vascular endothelial growth factor, suppression of inflammatory cell proliferation and migration, and increase in blood-retinal barrier function with edema resolution.

IVTA is a potent, long-acting steroid drug, capable of inhibiting inflammation, improving blood-retinal barrier status, and decreasing vascular permeability and leakage. These mechanisms may rapidly and significantly reduce macular thickness in CME secondary to RP [2, 34]. Intravitreal delivery enhances its performance and decreases systemic side effects. The main ocular complications with this route of administration are secondary glaucoma, cataract, and endophthalmitis [22]. Our patient experienced a significant decrease in retinal thickness together with a moderate improvement in BCVA and we observed worsening of the subcapsular cataract in the left eye. Furthermore, after one month of follow-up we observed a bilateral peak of intraocular pressure that was successfully controlled with topical timolol and brimonidine.

The improvement in visual acuity was not as marked as the reduction in retinal thickness or leakage. It is possible that this patient had reduced acuity due to foveal cell loss prior to the development of edema and/or irreversible functional damage due to edema. It is possible that the duration of CME and/or the stage of RP affect the prognosis of visual function in the case of patients with RP [2, 26, 35–39].

The left eye was treated first because of poorer visual acuity. Initially successful results encouraged treatment of the other eye, but the effect of IVTA was temporary and CME recurred 4 months after injection. In the right eye, we observed improvement in BCVA and reasonable anatomic results. Despite the recurrence of the CME, IVTA can be present intraocularly in measurable concentrations up to 1.5 years after intravitreal injection [40].

In an attempt to modulate this autoimmune process and the inflammatory mediators, some authors reported temporary improvement of CME in RP with administration of peribulbar steroids [28]. On this basis, we performed subtenon's triamcinolone in both our patient's eyes, which decreased retinal thickness in the right eye, but with no visual benefit. This form of administration could be associated with lower risk of complications related to intraocular procedure, such as endophthalmitis, and is less costly than intravitreal injection. Unfortunately, the subcapsular cataract in the left eye can interfere with the visual results. Additional studies are necessary to evaluate the frequency of repeated injections for these recurrences.

In conclusion, intravitreal and subtenon depot administration of triamcinolone may be useful for CME in patients with RP, but its efficacy seems to be limited over time and it is necessary to repeat the treatment after several months to maintain good anatomical results and improved BCVA. This visual acuity improvement permits better psychological and functional behaviour in patients with this type of disease. The subtenon depot triamcinolone can be safer and more cost-effective than intravitreal injection.

Although there is a poor correlation between change in visual acuity and decrease in retinal thickness or leakage in the macular area, it seems logical to maintain the retinal thickness as close to the “natural state” as possible.

Finally, careful observation over a longer period of time is essential in order to control potential complications related to the treatment.
